# Supplementary material for: Diversity and Phylogeny of Novel Cord-Forming Fungi from Borneo
Source: Microorganisms. 2022 Jan 22;10(2):239. doi: 10.3390/microorganisms10020239 (PMC8874581; doi:10.3390/microorganisms10020239)
Supplement: Supplementary file 1 [file microorganisms-10-00239-s001.zip › microorganisms-1530398-supplementary.pdf]

Supplementary Table S1.
Table showing the top 3 hits on UNITE with percentage match, e-value and sample country of origin. (-) denotes that sampling location is unknown.

BOR	Closest match - UNITE	Accession no	% ID	e-value	Origin
MN102414 BOR94	Cortinarius salor	UDB034759	82	0.0	Estonia
	Cortinarius pseudotriumphans	AY669600	82	4e-175	Chile
	Envir: Cortinarius	UDB004013	82	1e-174	Australia
MN102415 BOR82	Odonticium	JN710578	91	6e-180	Jamaica
	Junghuhnia micropora	JN710559	82	5e-175	Russia
	Atraporiella neotropica	HQ659221	81	5e-175	Belize
MN102416 BOR202	Gerronema subclavatum	U66434	88	0.0	-
	Gerronema	UDB016643	88	0.0	Gabon
	Gerronema nemorale	EU883594	87	0.0	Korea
MN102417 BOR203	Stereopsis	UDB033468	96	0.0	Laos
	Inflatostereum	AB971705	96	0.0	Okinawa, Japan
	Basidiomycota	UDB034476	95	0.0	Laos
MN102418 BOR83	Gerronema	UDB039651	99	0.0	Laos
	Gerronema nemorale	EU883594	92	0.0	Korea
	Gerronema subclavatum	U66434	92	0.0	-
MN102419 BOR110	Trametes	UDB013330	98	0.0	Malaysia
	Tinctoporellus	UDB039590	90	0.0	Laos
	Basidiomycota	UDB034554	90	0.0	Laos
MN102420 BOR205	Hyphodontia	UDB013979	85	0.0	Cameroon
	Envir: Eukaryota	UDB0762736	82	5e-175	Australia
	Globulicium hiemale	DQ873595	81	6e-174	Sweden
MN1024121 BOR100	Skeletocutis brevispora	UDB0799092	82	0.0	Estonia
	Skeletocutis	KY953055	81	0.0	China
	Skeletocutis nivea	KY953069	81	0.0	Indonesia
MN102422 BOR97	Polyporales	MG982516	78	2e-174	Tennessee, USA
	Incrustoporia	UDB0780441	76	2e-149	Brazil
	Skeletocutis nivea	KY953069	78	1e-138	Indonesia
MN102423 BOR91	Polyporales	MG982516	78	2e-174	Tennessee , USA
	Skeletocutis nivea	KY953069	78	5e-149	Indonesia
	Skeletocutis	KY953055	78	4e-138	China
MN102424 BOR101	Phallus hadriani	UDB022824	93	0.0	Latvia
	Phallus multicolor	KP012762	91	1e-174	Australia
	Phallus impudicus	UDB015413	92	4e-174	Estonia

MN102425 BOR77	Ceriporia sordescens	KX752606	92	0.0	USA
	Ceriporia sericea	KX752609	91	0.0	Russia
	Ceriporia	UDB0780147	90	0.0	India
MN102426 BOR96	Marasmius	KP013041	97	0.0	NT, Australia
	Marasmius brunneoaurantiacus	KX963788	95	0.0	Korea
	Marasmius	UDB039654	93	0.0	Laos
MN102427 BOR204	Marasmius	LC505241	91	0.0	Japan
	Marasmius	LC505255	90	0.0	Japan
	Uncultured fungi	JN890126	90	0.0	Guyana
MN102428 BOR90	Phanerochaete laevis	KP676125	96	0.0	India
	Phanerochaete affinis	EU118652	96	0.0	Sweden
	Hydnophlebia chrysorhiza	KP715570	95	0.0	India
MN102429 BOR105	Gerronema subclavatum	U66434	91	0.0	-
	Gerronema	UDB016643	90	0.0	Gabon
	Gerronema strombodes	KY242507	89	0.0	Tennessee, USA
MN102430 BOR80	Gerronema subclavatum	U66434	90	0.0	-
	Gerronema	UDB016643	92	0.0	Gabon
	Agaricales	UDB024521	89	0.0	Laos
MN102431 BOR98	Gerronema	UDB039651	98	0.0	Laos
	Gerronema kuruvense	UDB034480	97	0.0	Laos
	Gerronema strombodes	KY242507	91	0.0	Tennessee, USA
MN102432 BOR86	Trogia	UDB032694	99	4e-166	Laos
	Gerronema	UDB039651	99	4e-166	Laos
	Gerronema kuruvense	UDB032694	99	2e-164	Laos
MN102433 BOR102	Agaricales	UDB024521	99	0.0	Laos
	Gerronema subclavatum	U66434	86	0.0	-
	Gerronema nemorale	EU883594	86	0.0	Korea
MN102434 BOR88	Agaricales	UDB024521	100	0.0	Laos
	Gerronema	UDB039651	94	0.0	Laos
	Gerronema subclavatum	U66434	94	1e-179	-
MN102435 BOR81	Cystodermella granulosa	UDB0778329	98	0.0	British Columbia, Canada
	Cystodermella ambrosii	UDB034138	98	0.0	Finland
	Floccularia	MT756210	98	0.0	Arizona, USA

MN102436 BOR85	Psathyrella	UDB033876	99	0.0	Laos
	Envir: Eukaryota	UDB0755802	99	0.0	Colombia
	Psathyrella candolleana	MT340082	99	0.0	Arizona, USA
MN102437 BOR78	Trechispora	JF691276	98	0.0	Reunion
	Trechisporales	UDB014010	96	1e-173	Cameroon
	Trechispora regularis	AF347087	96	2e-170	Jamaica
MN102438 BOR201	Trechisporales	UDB014010	91	0.0	Cameroon
	Corticium confine	KP814404	91	0.0	Ontario, Canada
	Trechispora	AB520451	91	0.0	Japan
MN102439 BOR79	Trechisporales	UDB014010	90	0.0	Cameroon
	Trechispora	JF691276	90	0.0	Reunion
	Envir: Trechispora	UDB0779333	93	0.0	Sweden

Supplementary Table S2.
Table showing the top 3 hits on GenBank with values given for percentage sequence cover, percentage match, e-value and sample country of origin. (-) denotes that sampling location is unknown.

**BOR**	**Closest match – NCBI Genbank**	**Accession no**	**% cover**	**% ID**	**e-value**	**Origin**
MN102414 BOR94	Cortinarius delibutus	KY964801.1	100	84	6e-165	Minnesota, USA
	Cortinarius salor	AY669592.1	100	84	4e-162	-
	Uncultured Cortinarius	MK770312.1	100	83	5e-161	-
MN102415 BOR82	Odonticium sp.	JN710578.1	65	91	0.0	Jamaica
	Trechispora subhelvetica	JN710601.1	100	83	7e-165	Norway
	Atraporiella neotropica	HQ659221.1	100	82	1e-156	Belize
MN102416 BOR202	Gerronema subclavatum	U66434.1	100	88	0.0	-
	Gerronema nemorale	EU883594.1	100	88	0.0	Korea
	Gerronema sp.	JQ657797.1	100	88	0.0	Gabon
MN102417 BOR203	Inflatostereum aff. glabrum	AB971705.1	94	96	0.0	Okinawa, Japan
	Gerronema subclavatum	U66434.1	100	92	0.0	-
	Gerronema sp.	JQ657797.1	100	92	0.0	Gabon
MN102418 BOR83	Gerronema nemorale	EU883594.1	100	92	0.0	Korea
	Gerronema subclavatum	U66434.1	100	92	0.0	-
	Gerronema sp.	JQ657797.1	99	91	0.0	Gabon
MN102419 BOR110	Uncultured Polyporales	GQ268623.1	80	94	0.0	Malaysia (Borneo)
	Trametes sp.	KJ832023.1	78	90	0.0	Peru
	Trametes elegans	OL684960.1	78	90	0.0	-
MN102420	Rickenella sp.	MT537051.1	100	82	2e-159	Australia

BOR205						
	Uncultured fungus	JX316513.1	100	82	2e-159	Argentina
	Globulicium hiemale	DQ873595.1	99	81	1e-152	Sweden
MN1024121 BOR100	Skeletocutis delicata	MF685355.1	78	95	2e-155	Estonia
	Skeletocutis exilis	MF685359.1	78	95	4e-153	Estonia
	Lenzites betulinus	MT644927.1	63	94	3e-154	Denmark
MN102422 BOR97	Skeletocutis bambusicola	MN908950.1	68	86	2e-110	Thailand
	Polyporaceae sp.	KP013024.1	60	86	1e-107	NT, Australia
	Uncultured Skeletocutis	HM136669.1	58	87	1e-102	-
MN102423 BOR91	Skeletocutis bambusicola	MN908950.1	71	86	2e-110	Thailand
	Polyporaceae sp.	KP013024.1	63	86	1e-107	NT, Australia
	Uncultured Skeletocutis	HM136669.1	61	87	1e-102	-
MN102424 BOR101	Phallus multicolor	KP012762.1	82	98	5e-174	Australia
	Phallus impudicus	MT512648.1	83	96	7e-168	-
	Phallus aff. rubicundus	KP012967.1	82	96	9e-167	Australia
MN102425 BOR77	Ceriporia sordescens	KX752606.1	94	94	0.0	USA
	Ceriporia sericea	KX752609.1	94	92	0.0	Russia
	Meruliopsis taxicola	EU118648.1	93	91	0.0	Sweden
MN102426 BOR96	Marasmius sp.	KP013041.1	100	97	0.0	Australia
	Marasmius brunneoaurantiacus	KX963788.1	100	94	0.0	Korea
	Uncultured fungus	JN889907.1	100	94	0.0	Guyana
MN102427 BOR204	Uncultured Ascomycota clone	EU490128.1	100	90.62	0.0	USA
	Marasmius chrysocephalus	MN930586.1	79	92.55	0.0	Guyana
	Marasmius sp.	MN930555.1	76	91.81	0.0	Guyana
MN102428 BOR90	Phanerochaete affinis	EU118652.1	100	96.01	0.0	Sweden
	Phanerochaete laevis	KP676125.1	100	95.99	0.0	India
	Phanerochaete luteoaurantiaca	MT537067.1	100	95.43	0.0	Perth, Australia
MN102429 BOR105	Gerronema subclavatum	U66434.1	100	92	0.0	-
	Gerronema sp.	JQ657797.1	100	91	0.0	Gabon
	Gerronema strombodes	KY242507.1	100	90	0.0	Tennessee, USA
MN102430 BOR80	Gerronema subclavatum	U66434.1	94	92	0.0	-
	Gerronema sp.	JQ657797.1	94	92	0.0	Gabon
	Gerronema strombodes	KY242507.1	100	88	0.0	Tennessee, USA
MN102431 BOR98	Gerronema strombodes	KY242507.1	92	93	0.0	Tennessee, USA
	Gerronema subclavatum	U66434.1	91	93	0.0	-
	Gerronema sp.	JQ657797.1	91	93	0.0	Gabon

MN102432 BOR86	Gerronema subclavatum	U66434.1	99	99	3e-165	-
	Gerronema sp.	JQ657797.1	98	99	4e-164	Gabon
	Megacollybia fallax	MT437075.1	99	99	1e-163	Arizona, USA
MN102433 BOR102	Trogia infundibuliformis	KP794601.1	88	90	0.0	Sri Lanka
	Gerronema sp.	JQ657797.1	92	89	0.0	Gabon
	Gerronema subclavatum	U66434.1	87	90	0.0	-
MN102434 BOR88	Gerronema subclavatum	U66434.1	100	94	2e-178	-
	Gerronema sp.	JQ657797.1	100	94	1e-175	Gabon
	Megacollybia platyphylla	LT854019.1	100	93	7e-173	Czech Republic
MN102435 BOR81	Floccularia cf. luteovirens	MT756210.1	98	98	0.0	Arizona, USA
	Cystodermella cinnabarina	OK491631.1	98	98	0.0	Arizona, USA
	Stropharia caerulea	MT644935.1	97	98	0.0	Denmark
MN102436 BOR85	Psathyrella cf. candolleana	MT424873.1	100	99	0.0	Arizona, USA
	Psathyrella badhyzensis	KC992883.1	100	99	0.0	-
	Psathyrella trinitatensis	KC992882.1	100	99	0.0	-
MN102437 BOR78	Uncultured Trechisporales	JF691276.1	96	98	4e-174	Reunion
	Trechispora regularis	AF347087.1	99	96	5e-169	-
	Uncultured ectomycorrhizal fungus	KT461299.1	96	96	5e-164	Democratic Republic of Congo
MN102438 BOR201	Uncultured Trechisporales	JF691276.1	91	92	0.0	Reunion
	Uncultured fungus	AB520451.1	90	91	0.0	Japan
	Corticium confine	KP814404.1	90	91	0.0	Ontario, Canada
MN102439 BOR79	Uncultured Trechisporales	JF691276.1	98	91	0.0	Reunion
	Uncultured fungus	AB520451.1	85	93	0.0	Japan
	Trechispora hymenocystis	MT816397.1	85	92	0.0	Norway
